# Sulphated Polysaccharides from *Ulva clathrata* and *Cladosiphon okamuranus* Seaweeds both Inhibit Viral Attachment/Entry and Cell-Cell Fusion, in NDV Infection

**DOI:** 10.3390/md13020697

**Published:** 2015-01-26

**Authors:** José Alberto Aguilar-Briseño, Lucia Elizabeth Cruz-Suarez, Jean-François Sassi, Denis Ricque-Marie, Pablo Zapata-Benavides, Edgar Mendoza-Gamboa, Cristina Rodríguez-Padilla, Laura María Trejo-Avila

**Affiliations:** 1 Laboratorio de Inmunología y Virología, Facultad de Ciencias Biológicas, Universidad Autónoma de Nuevo León, Av. Manuel L. Barragán y Av. Pedro de Alba s/n Cd. Universitaria, San Nicolás de los Garza, N.L. 66455, Mexico; E-Mails: albertvirus@gmail.com (J.A.A.-B.); lucia.cruzsr@uanl.edu.mx (L.E.C.-S.); denis.ricquemr@uanl.edu.mx (D.R.-M.); paablo.zapatabn@uanl.edu.mx (P.Z.-B.); edgar.mendozagm@uanl.edu.mx (E.M.-G.); cristina.rodriguezpd@uanl.edu.mx (C.R.-P.); 2 Centre d’Etude et de Valorisation des Algues, Presqu'île de Pen Lan, 22610 Pleubian, France; Present Address: CEA Cadarache, Algae Processes and Technologies, 13108 St Paul Lez Durance, France; E-Mail: Jean-Francois.SASSI@cea.fr

**Keywords:** antiviral, seaweeds, sulphated polysaccharides, *Ulva clathrata*, *Cladosiphon okamuranus*, Newcastle Disease Virus

## Abstract

Sulphated polysaccharides (SP) extracted from seaweeds have antiviral properties and are much less cytotoxic than conventional drugs, but little is known about their mode of action. Combination antiviral chemotherapy may offer advantages over single agent therapy, increasing efficiency, potency and delaying the emergence of resistant virus. The paramyxoviridae family includes pathogens causing morbidity and mortality worldwide in humans and animals, such as the Newcastle Disease Virus (NDV) in poultry. This study aims at determining the antiviral activity and mechanism of action *in vitro* of an ulvan (SP from the green seaweed *Ulva clathrata*), and of its mixture with a fucoidan (SP from *Cladosiphon okamuranus*), against La Sota NDV strain. The ulvan antiviral activity was tested using syncytia formation, exhibiting an IC_50_ of 0.1 μg/mL; ulvan had a better anti cell-cell spread effect than that previously shown for fucoidan, and inhibited cell-cell fusion via a direct effect on the F0 protein, but did not show any virucidal effect. The mixture of ulvan and fucoidan showed a greater anti-spread effect than SPs alone, but ulvan antagonizes the effect of fucoidan on the viral attachment/entry. Both SPs may be promising antivirals against paramyxovirus infection but their mixture has no clear synergistic advantage.

## 1. Introduction

Since the first report of the antiviral activity of sulphated polysaccharides in 1958 [1], a large number of SP have been found to possess a broad spectrum of antiviral activity. Nowadays a number of these SP have been demonstrated to be potent inhibitors of paramyxoviruses, including parainfluenza virus, respiratory syncytial virus, mumps virus, measles virus, NDV, and distemper canine virus [2,3,4]. Ulvales (chlorophyta) are seaweeds distributed worldwide. The genera *Ulva*, before *Enteromorpha*, is grown and collected for food consumption [5,6]. Among the polymers synthetized by these *Ulva* sp., cell wall polysaccharides represents 38%–54% of all dry algal matter [7], and can be classified in four polysaccharide families: two major ones, ulvan and cellulose and two minor ones, xyloglucan and glucurunan. Ulvan, the most important one, has been characterized with a variety of biological features [8,9,10,11,12,13,14]; among these, the immunomodulatory effect and the antiviral activity have focused the attention of scientists worldwide and marked a trend in drugs development. The main constituents reported in ulvans from several Ulvales species [15,16,17,18,19] are: Rhamnose (16.8%–45.0% dw), xylose (2.1%–12.0%), glucose (0.5%–6.4%), uronic acid (6.5%–19.0%), sulphate (16.0%–23.2%) and iduronic acid (1.1%–9.1%), rhamnose occurring mainly in the form of the aldobiouronic acid, 4-*O*-β-d-glucuronosyl-l-rhamnose.

Newcastle disease virus (NDV) is the causative agent of one of the most serious threats to the poultry industry and causes enormous economic losses because of its worldwide distribution and high flock mortality [20]. It belongs to the Paramyxoviridae family, which groups enveloped, negative-sense single-stranded RNA viruses [21], and utilizes its genome to code for six proteins from the 3′ terminus to the 5′ terminus [22]. The viral lipid envelope contains the viral glycoproteins HN (attachment protein) and F (fusion protein), both being needed for efficient infection of cells; the F glycoprotein mediates the penetration of the cellular membrane during viral entry [22]. Currently there are no antiviral agents available against NDV in poultry, hence the importance of developing new alternative control measures.

In our study, we evaluated the anti-NDV activity and mechanism of action of the SP ulvan (isolated from the green algae *Ulva clathrata*) and of a mixture with *Cladosiphon okamuranus* fucoidan, which mode of action has previously been characterized [3,4], for the purpose of developing new candidates anti- paramyxovirus drugs.

## 2. Results and Discussion

### 2.1. Composition of Ulva Clathrata Powder and Ulvan Extract

**Figure 1:**
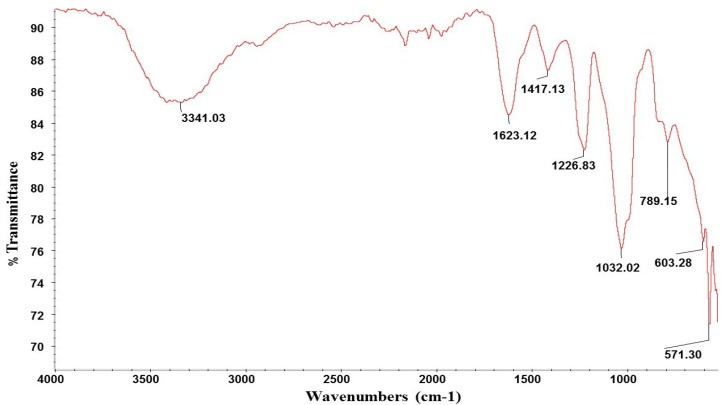
Infrared absorption spectrum of the native ulvan from *Ulva clathrata*.

**Figure 2:**
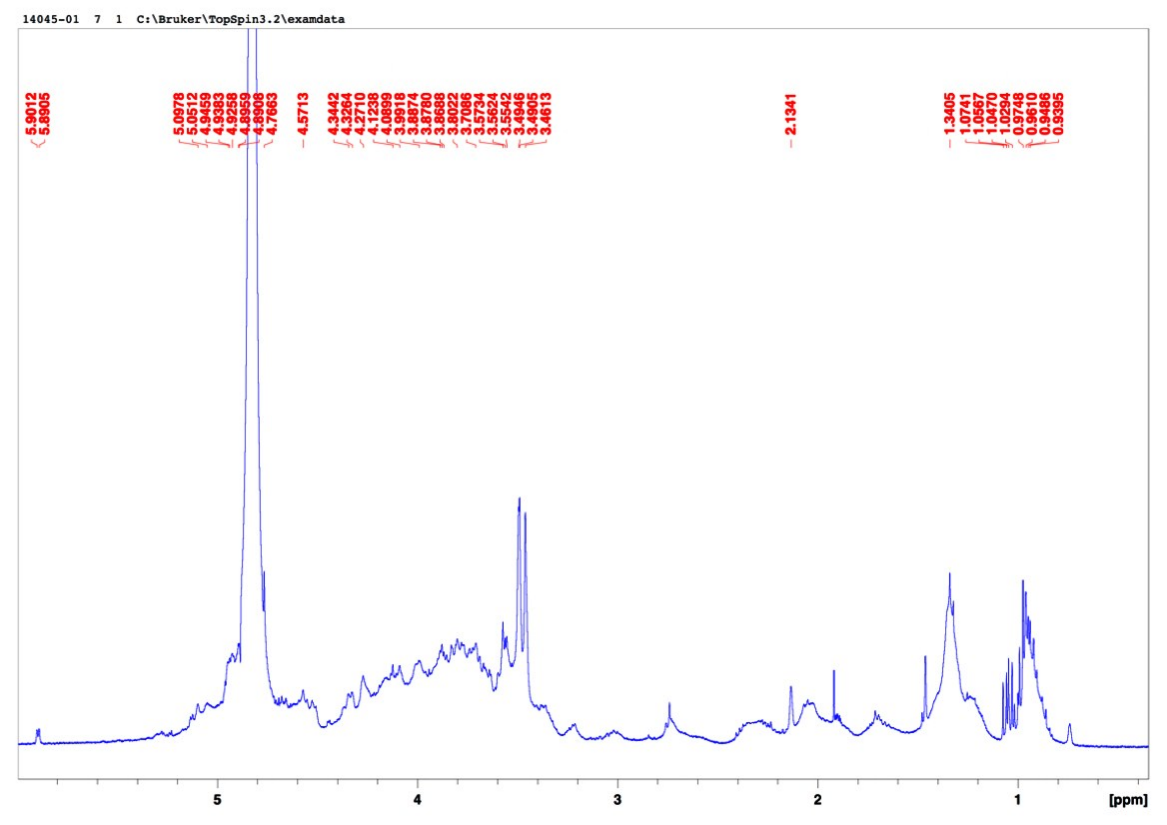
^1^H-NMR spectrum in D_2_O of the native ulvan from *Ulva clathrata*.

The composition of the starting *Ulva clathrata* powder was 22.4% ash, 15.3% protein, 9.5% sulphate, 11.4% rhamnose, 7.3% glucuronic acid, 3.0% xylose, 2.5% glucose and 1.4% galactose. The final composition of the ulvan extract was 19% ash, 10.0% protein, 11.0% sulphate, 21.7% rhamnose, 13.9% glucuronic acid, 6.1% glucose, 5.0% xylose, 3.6% iduronic acid, and 2.2% galactose. The molecular weight was 359,800 g·mol^−1^ equivalent pullulan. *Ulva* sp. powder is known to contain high amounts of good-quality protein, carbohydrate, vitamins and minerals [23]. *Ulva clathrata* chemical composition found in present study is on the line with that reported by Peña-Rodriguez *et al.* [15] for the same Ulva species. Ulvan composition has been widely studied [7,19] and showed to vary according to several factors including the period of collection, the ecophysiological growth conditions, the taxonomic origins and the post-collection treatment of the algal sources [7]. Extraction is conventionally achieved by using warm water (80–90 °C) containing ammonium oxalate as a divalent cation chelator, and the recovery of ulvan is generally obtained by precipitation in ethanol. As ulvan precipitation with ethanol is not specific, the extraction and purification are not completely effective in the removal of cellulose, hemicellulose, proteins and ash [24]. The final step for removing contaminants may vary considerably. Ulvan is mainly built on disaccharides repeating sequences composed of sulphated rhamnose and glucuronic acid, iduronic acid or xylose. Low proportions of galactose, glucose and protein are also generally found in ulvan [7,19,25]. The sugar composition of the purified *U. clathrata* ulvan obtained in present study is very similar to that given by Quemener *et al.* [25] for an *U. lactuca* ulvan that contained rhamnose 20.8%, glucuronic acid 16.0%, iduronic acid 3.7%, xylose 3.5%, glucose 2.8%, and galactose 0.7%; this *Ulva lactuca* ulvan also contained protein 11.6% and sulphate 21.2%. These values are congruent with those reported for ulvan from other origins [26,27,28]. The polysaccharide extracted from *U. clathrata* in present study was more sulphated than those reported for *Ulva armoricana* (10.3%–13.8%) and *Ulva rotundata* (9.2%–12.5%) [16,29] but similar to those obtained from *Ulva conglobata* (23.04%–35.2%) [14], and less sulphated than the *Ulva clathrata* ulvan reported by Hernandez-Garibay *et al.* [30]. Ulvan PS characterization was completed by FT-IR and H-NMR spectra: the FT-IR spectrum (Figure 1) is comparable with the previously published data for *Ulva rigida* and *Ulva pertusa* polysaccharides [28,31]. A characteristic band at 3341.03 cm-1 corresponding to -OH groups, which is consistent with the structure of disaccharides is observed. Two bands at 1226.83 cm^−1^ and 789 cm^−1^ are indicatives to the bounds C-O-S of sulfated groups. The band 1623 cm^−1^ is allocated to the vibration of C=O of the uronic acid; H-NMR spectrum (Figure 2) is comparable with data for ulvans [32]. All aromatic protons bound to -OH groups are in the region of 4.5 to 5.0. Protons attached to ether groups are allocated in the region of 3.4 to 3.6. Methyl protons of rhamnose are assigned in the region of 0.8 to 1.1.

### 2.2. Cytotoxicity of the SP

**Figure 3:**
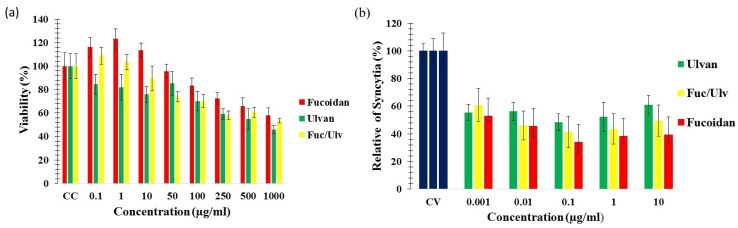
Cytotoxicity and antiviral activity of sulphated polysaccharides (SP) against Newcastle Disease Virus (NDV). (**a**) Vero cells (6.5 × 10^3^ cells), seeded in 96-well plates, were treated with different concentrations of SP for 48 h. Cell viability was then determined in triplicates by the MTT assay and compared with that in untreated cells (100% viability); (**b**) Vero cells (6.7 × 10^4^ cells), seeded in 24-well plates, were infected with NDV and treated with different concentrations of SP during infection for 1 h, and the antiviral activity was determined in a syncytia reduction assay. The data shown are the mean ± standard deviation (SD) of triplicate cultures, and the experiment was repeated three times. CC, cellular control; CV, viral control.

To determine the cytotoxicity of SP and their mixture, MTT assays were performed. No significant cytotoxicity was detected for the SP at concentrations up to 100 μg/mL in Vero cells; the 50% cytotoxic concentrations (CC_50_) for fucoidan, ulvan and the mixture were of 1336 μg/mL, 810 μg/mL and 823 μg/mL, respectively (Figure 3a, Table 1). This is consistent with studies that have assessed the cytotoxicity of other SP in cell culture, showing lower toxicity than commercial, FDA-approved, antiviral drugs [3,8,33].

**Table 1:** Antiviral activity and cytotoxicity of sulphated polysaccharides.

Polysaccharide	Vero Cell Line		
	CC_50_ (μg/mL) ^a^	IC_50_ (μg/mL) ^b^	SI ^c^
Fucoidan	1136	0.01	113,633
Ulvan	810	0.1	8102.5
Fuc/Ulv	823	0.01	82,325

^a^ Concentration of test compound (μg/mL) that reduced Vero cell viability by 50%. ^b^ Concentration of a test compound that reduced the number of NDV syncytia in Vero cells by 50%. ^c^ Values of selectivity index.

### 2.3. Antiviral Activity in Vitro

The antiviral activity of the SP against NDV was evaluated by syncytia reduction assays. Vero cell monolayers were treated with different concentrations below or equal to 100 μg/mL of the ulvan, fucoidan and their mixture [1:1] at the time of infection with 1 × 10^5.5^ TCID_50_ of NDV, and the concentration of the SP was maintained throughout the infection. As shown in Figure 3b, a concentration-dependent inhibition of viral entry into the host cells was observed with the addition of the SP compared to the findings in the untreated control cells. The results showed that the SP inhibits syncytia formation, with 50% inhibitory concentrations (IC_50_) values of 0.01 μg/mL, 0.1 μg/mL and 0.01 μg/mL for fucoidan, ulvan and the mixture, respectively.

The selectivity index (SI_50_) of the compounds was calculated to be >100,000, >8000 and >80,000 for fucoidan, ulvan and their mixture, respectively. Fucoidan showed the most potent antiviral activity at a concentration of 0.1 μg/mL, inhibiting by 66% the syncytia formation; ulvan and the mixture at 0.1 μg/mL inhibited syncytia by 51.54% and 58.67%, respectively (Table 1).

Although fucoidan showed a better antiviral activity than ulvan, ulvan itself exhibited a good ability to inhibit syncytia formation. Similar results were obtained previously with brown algae SP and green algae ulvans against enveloped viruses [3,4,10,34,35]. There is just one report that correlates the chemical structure of ulvan with its antiviral activity [35]; the authors attributed the antiviral activity of *U. lactuca* ulvan to its high molecular weight (MW) based on the work of Lee *et al.* [36], who reported that SP with higher MW have better antiviral activities. In our previous report [3] we assessed the antiviral activity of *C. okamuranus* fucoidan, which has a significant content of glucuronic acid (23%) and low levels of sulphatation (9.6%). *U. clathrata* ulvan studied in present work has a higher level of sulphatation (11%) and a lower level of glucuronic acid (13.9%). More reports are needed in order to correlate the chemical structure of ulvans with their antiviral effect.

**Figure 4:**
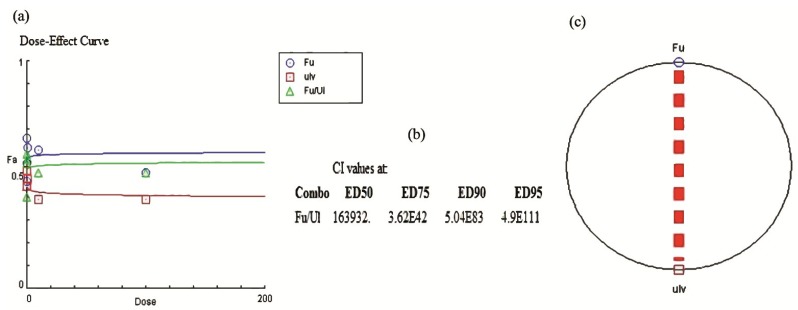
Dose-effect relationships of fucoidan and ulvan, alone or in combination. (**a**) A dose-effect curve based on data obtained in syncytia reduction assay, the curve depicts no sigmoidal (synergism) or plateau effect (summation) shapes indicating an antagonism relationship between the SP; (**b**) Combination Index (CI) values of the mixture of SP at different Effective Doses (ED) based on syncytia reduction assay and calculated by the median-effect method of Chou and Talalay. CI values > 1 indicates an antagonism effect; (**c**) A polygonogram plot indicating a very strong antagonism (very thick dash line) between the SP.

We apparently observed a decrease of the antiviral activity for the mixture in comparison with fucoidan alone, probably by a kind of antagonism. Our results agree with a previous report [3] where we determined the antiviral activity and mechanism of action of *C. okamuranus* fucoidan against NDV, demonstrating the powerful antiviral activity of fucoidan. We expected a greater effect from the mixture. Combination chemotherapy with synergistically active antiviral agents that have different targets from each other for viral replication may offer several advantages over single agent therapy, such as greater potency, superior clinical efficacy, reduction of toxicity and side effects due to the reduction of the drug dosage needed, suppression of the emergency of drug—resistant viruses, and greater cost effectiveness [37]. Hayashi *et al.* [37] evaluated the effect of the *Undaria pinnatifida* fucoidan and the FDA-approved drug oseltamivir against influenza H1N1 virus; they observed a synergistic effect of the compounds combination due to different targets of their tested compounds on the H1N1 replication cycle and determined this synergistic effect by an isolobologram. The conjugates of κ-carrageenans and AZT (3′-azido-3′-deoxythymidine) exert synergistic effects on the reduction of the infection of the human immunodeficiency virus (HIV) [38]. In contrast, Takebe *et al.* [39] isolated lectins from red and blue-green algae and concluded that the combination of the algae-derived proteins with IFN-α did not exert a synergistic or additive effect, leading us to conclude that sulphated polysaccharides have special features that could explain their potent antiviral effect. Since there is nowadays no FDA approved antiviral drug for NDV, and therefore no possibility to mix our biological metabolite with a commercial drug, we determined the antiviral effect of the mixture of two polysaccharides isolated from two different algae; we expected a synergism or an additive effect with the mixture; instead of this, the mixture showed a decrease in the antiviral activity. We determined the combination effect of the mixture with the median effect method described by Chou and Talay [40,41] (Figure 4). Figure 4a depicts the dose-effect relationship of the compounds: for a synergistic effect we could expect a sigmoidal plot, but we observed a linear plot. The combination index values (CI) (Figure 4b) determined at different effective doses by the median effect method, showed that the mixture have a strongest antagonism, CI > I indicates antagonism; we corroborated these results with the polygonogram (Figure 4c) where we can see a very thick dash line characteristic of the antagonist effect. We suggest that this antagonism is due to simultaneous action of both SP on the same target. The median effect method has been used in many reports to assess the combo effect of antiviral drugs [42,43,44].

### 2.4. Virucidal Activity of Ulvan

Mendes *et al.* [10] demonstrated that algae extracts from *Ulva fasciata* have the possibility to inactivate virions. To analyse the possibility that *U. clathrata* ulvan acts directly on the virus particle, leading to infectivity inactivation, a virucidal assay against NDV virions was conducted. Ulvan did not exert virucidal effect against NDV virions at the tested concentrations, indicating that the inhibitory effect detected by the syncytia reduction assay was actually due to an interference with some step of the NDV replication cycle (Table 2). The lack of virucidal activity is in agreement with previous studies with the *C. okamuranus* fucoidan [3,33] and other pure algae SP that cannot induce significant virions inactivation [45,46,47].

**Table 2:** Virucidal assay based in the presence or absence of the cytopathic effect of NDV.

		Time	Viral Titer	
			1 × 10^4^ TCID_50_	1 × 10^5^ TCID_50_
	CC		―	―
	CV		+++	+++
**Ulvan concentrations**	10 μg/mL	0 h	+++	+++
	10 μg/mL	3 h	+++	+++
	10 μg/mL	6 h	+++	+++
	100 μg/mL	0 h	+++	+++
	100 μg/mL	3 h	+++	+++
	100 μg/mL	6 h	+++	+++

### 2.5. Ability of the SP to Block NDV-Induced Cell-Cell Fusion

**Figure 5:**
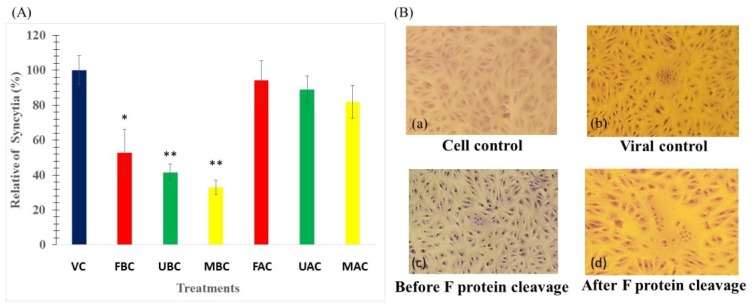
Inhibition of an avirulent NDV (La Sota) strain-mediated cell fusion. (**A**) Fusion inhibition assay with SP and their mixture treatment before, before cleavage [BC] and after F protein cleavage, after cleavage [AC]. Data are expressed as the percent of the number of syncytia compared with the findings in virus-infected control cells. VC, viral control; F, fucoidan; U, ulvan; M, mixture. The data shown are the mean ± SD of triplicate experiments. The asterisks indicate a significant difference between the treatment and viral control (* *p* < 0.05 and ** *p* < 0.001); (**B**) Vero cells infected with NDV and treated by trypsin digestion: (**a**) Uninfected control cells (100×); (**b**) Vero cells infected with NDV and treated with trypsin (100×); (**c**) Infected Vero cells were treated with 0.1 μg/mL of the SP and their mixture before F protein cleavage, at which point syncytia formation could be inhibited if SP were added (100×); (**d**) Infected Vero cells were treated with SP and their mixture after F protein cleavage, and the SP could not inhibit syncytia formation in this condition (100×).

To determine whether the compound inhibits the cell-cell spread of NDV, we performed cell fusion inhibition assays as described in the experimental section. The NDV envelope contains two proteins related to entry: the attachment protein HN and the fusion protein F. After one replication cycle, both glycoproteins are displayed in the cell envelope. Fusion of an infected cell with a non-infected occurs due to the recognition of sialic acid by HN and a conformational change of F protein, driving to syncytia formation, the characteristic cytopathic effect of NDV. Avirulent strains of NDV are characterized by their inability to form syncytia because the F0 protein cannot be cleaved in F1/F2. However, after trypsin digestion, syncytia formation was observed. Our results show that the SP and their mixture inhibited syncytia formation only if they were added before F protein cleavage (before trypsin digestion; Figure 5B). Before F protein cleavage, the mixture inhibited syncytia formation by 67% and reduced the syncytia size, compared with the findings in untreated viral-infected control cells, while fucoidan and ulvan inhibited syncytia formation by 47% and 59% respectively; therefore ulvan and the mixture apparently have a better anti-NDV cell-cell fusion spread than fucoidan alone. However, after F protein cleavage via trypsin digestion, the SP and their mixture lost the ability to inhibit syncytia formation (Figure 5A). This suggests that ulvan inhibits viral fusion by interacting with the intact F0 protein but not with the mature protein, as previously found with fucoidan [3], and that ulvan has the same mechanism of action as fucoidan.

Our results agree with other reports about algae SP being able to exert the ability to inhibit the cell-cell fusion [48]. Nyberg *et al.* [49] demonstrated that the yeast-derived SP PI-88 could inhibit the cell-cell spread fusion and consequent syncytia formation by HSV in GMK AH1 cells. Cagno *et al.* [50] reported the anti-cell-cell spread fusion of SP derived from *Escherichia coli* of RSV; they observed a considerable reduction of syncytia inHep-2 and A549 cells. Thus SP from different sources could be powerful inhibitors of viral cell-cell fusion.

## 3. Experimental Section

### 3.1. Antiviral Agents

#### 3.1.1. Fucoidan from *Cladosiphon okamuranus*

Fucoidan was purchased as a dried powder from Kadoya & Co., Kobe, Japan (lot A03012), extracted (as described by Tako *et al.* 2000 [51] from cultured kelp *Cladosiphon okamuranus* harvested off the coast of Okinawa Island, Japan. The polysaccharide preparation was certified to contain 90.4% fucoidan (anthrone-sulfuric acid method) and exhibit a mean molecular weight of 92.1 kDa (size exclusion HPLC method). The fucose and sulphate content of fucoidan were of 38.6% and 15.9%, respectively, with ash comprising 9.6% of the content and other sugars comprising 23% (glucuronic acid and traces of xylose). Dried samples of fucoidan were suspended in Dulbecco’s modified Eagle’s medium (DMEM) at a concentration of 2.5 mg/mL and filtered through a membrane filter (pore size, 0.22 mm).

#### 3.1.2. *Ulva clathrata* Culture

*Ulva clathrata* biomass was cultivated at Los Mochis, Sinaloa, México, under a patented technology developed for high yield and low cost production, from a clonal laboratory stock of an *U. clathrata* known strain [52]. Ulva sample was collected as described in Peña-Rodriguez *et al.* [15] from the pooled production of two shrimp culture ponds (7800 and 8400 m^2^) after a two month growing period (1 March–6 May 2008), with temperatures at 8:00 and 16:00 in ranges of 18–27 and 21–32 °C; initial algal biomass density was 11–12 g/m^2^ and reached 2 kg fresh weight/m^2^. The Ulva fresh biomass was washed in freshwater to remove sediment, and epiphytes, it was then pressed and dried at 50 °C in an industrial hot air dryer, and ground in the same industrial plant. The chemical composition of this sample was reported by Peña-Rodriguez *et al.* [15] as the large scale system (LSS) production.

#### 3.1.3. Extraction of Ulvan

A sample of dried *Ulva clathrata* was extracted at CEVA, France according to the procedure described by Robic *et al.* [16]. Dry ground seaweed tissue was soaked and stirred for 3 days at room temperature in 78% ethanol and recovered by filtration on a 100-μm sieve. The wet, depigmented cake of ground seaweeds was rinsed with deionized water and was extracted and purified by tangential ultrafiltration as described by Robic *et al.* [16]. Dried samples of ulvan were suspended in Dulbecco’s modified Eagle’s medium (DMEM) at a concentration of 2.5 mg/mL and filtered through a membrane filter (pore size, 0.22 mm).

#### 3.1.4. Characterization of *Ulva clathrata* Powder and Ulvan Extract

Dry solid content was determined from the loss in weight of samples kept for 24 h at 103 °C in a ventilated furnace. Ash content was quantified gravimetrically after 12 h at 550 °C. Sulphate content was measured according to Tabatabai [17]. Protein content was estimated using the Kjeldahl nitrogen method [53]. Neutral sugar and uronic acid composition was determined after methanolysis in MeOH-HCl and HPLC analysis using an Absorbosphere RP18 column (5 μm, 4 mm × 250 mm, Grace, Deerfield, IL, USA) according to Quemener *et al.* [18]. Molecular weight of the ulvan was determined as described by Robic *et al.* [29]: briefly, ulvan was injected on a high-performance size exclusion chromatography (HPSEC) system (Agilent 1100 Series HPLC Value System, Santa Clara, CA, USA) constituted of a 8 × 300 mm Shodex OHpak SB-806M HQ column (Showa Denko KK, Miniato, Japan) and a 6 × 50 mm Shodex OHpak SB-G guard column (Showa Denko KK, Miniato, Japan) eluted at 0.7 mL.min^-1^ with 50 mM NaNO_3_ containing 0.02% NaN; on-line molar mass determinations were performed using a multi-angle laser light scattering (MALLS) detector (mini-Dawn Wyatt, Santa Barbara, CA, USA) operating at three angles: 41°, 90° and 138° and a differential refractometer (ERC 7517A; Erma Inc., Tokyo, Japan), and a UV detector at 280 nm (LDC ⁄ Milton Roy SpectroMonitor 3000; LDC S.A., Paris, France)). Average molecular weight was calculated using ASTRA 1.4 software (Wyatt, Santa Barbara, CA, USA). The FTIR spectra of dried and ground algal material were recorded using a Thermo Scientific Nicolet 380 FT-IR spectrometer (Thermo Electron Scientific Instruments LLC, Madison, WI, USA) equipped with a Diamond ATR sampling device (Thermo Electron Scientific Instruments LLC, Madison, WI, USA), and using ASTM E204 protocols. For NMR analysis, 70 mg of sample were dissolved in D_2_O, then lyophilized, and dissolved again in D_2_O with 3-(trimethylsilyl)-propionic-2,2,3,3-*d*_4_ acid, sodium salt as zero reference. The NMR spectra were recorded at 25 °C using a Bruker Avance DPX 400 spectrometer (Bruker Mexicana, Mexico city, Mexico) equipped with gradients and a 5 mm multinuclear probe at base frequency of 100 MHz for ^13^C and 400 MHz for ^1^H.

### 3.2. Cell Line and VIRUS

Green African monkey kidney (Vero) cells were grown in DMEM/FL-12 supplemented with 5% (v/v) fetal bovine serum and 1% (v/v) antibiotics. The flasks were maintained in a humidified atmosphere with 5% CO_2_ at 37 °C. The La Sota NDV strain was obtained from FORT DODGE^®^ (Pfizer, New York, NY, USA) and tittered by fifty percent tissue culture infectious dose (TCID50) assay, according to their CPE effect as described by Reed and Muench [54].

### 3.3. Cytotoxicity Assay

To evaluate the cytotoxicity of the compounds MTT’s assays were performed. Vero cells were seeded in 96-well plates at an initial density of 7 × 10^3^ cells per well. The cells were incubated with increasing concentrations of the SP diluted in DMEM, for 48 h at 37 °C and 5% CO_2_. MTT (3-(4,5-dimethylthiazol-2-yl)-2,5-diphenyltetrazolium bromide; 0.5 mg/mL; 40 μL/well) was added to the cells, which were further incubated for 1 h 15 min. MTT was removed, and dimethylsulfoxide (DMSO; 100 μL/well) was added to dissolve the formazan crystals. The optical density of the cells was measured at 570 nm (Synergy™ HT multi-mode microplatereader; BIOTEK instruments Inc., Winooski, VT, USA). Each experiment was performed in triplicate, and experiments were repeated at least three times. The cytotoxicity was expressed as the CC_50_, which was the concentration of the test substances that inhibited the growth Vero cells by 50% compared with the growth of the untreated cells.

### 3.4. Virucidal Assay

The virucidal activity of ulvan against La Sota NDV was assessed using syncytia reduction assays with monolayers of Vero cells grown in 24-well plates. The assays where performed by adding the SP (0, 10, or 100 μg/mL) to an equal volume to NDV (1 × 10^4^ and 1 × 10^5^ TCID_50_). After 0, 3 and 6 h, the mixtures were added to Vero cells and further incubated 1 h at 37 °C. Thereafter, the mixtures were removed and DMEM with no serum and antibiotics was added. Because La Sota NDV is an avirulent strain, trypsin (0.001%; GIBCO) was added after 24 h to mediate syncytia formation after proteolytic cleavage of F0 protein and incubated for 30 min at 37 °C, afterwards trypsin was removed and DMEM was added. Monolayers were fixed with methanol:acetone (3:1) then incubated for 24 h at 37 °C and 5% CO_2_ and stained with 1% crystal violet in an incubator, after which syncytia presence or absence was evaluated.

### 3.5. Antiviral Activity

The antiviral activity of the SP and their mixture against La Sota NDV was assessed using syncytia reduction assays with monolayers of Vero cells grown in 24 well plates. The assays were performed treating with the SP (0.001–10 μg/mL) and infecting the cells (1 × 10^5.5^ TCID_50_ of NDV) at the same time for 1 h at 37 °C, allowing the virus to adsorb. The residual inoculum was discarded and the cells were washed with PBS, after which DMEM was added to the cells. Thereafter, monolayers were treated with trypsin (0.001%; GIBCO) for 30 min at 37 °C to allow F0 protein cleavage. Each concentration was investigated using three culture wells per each SP and their mixture concentration per experiment, the experiments were repeated at least three times. Monolayers were fixed with methanol:acetone (3:1) after incubation for 24 h at 37 °C in a 5% CO_2_ incubator and stained with 1% crystal violet, and subsequently syncytia were counted. By reference to the number of syncytia observed in viral control monolayers (untreated cultures), the IC_50_ was determined from dose-response curves. The median effect method [41] was used to determined synergism, antagonism or additive effect in the SP mixture.

### 3.6. Fusion Inhibition Assay

To determine if the SP has the possibility to inhibit cell-cell spread of NDV, Fusion inhibition assays were performed according to the method reported by Zhu *et al.* [55]. Vero cell monolayers in 24-well plates were infected with 1 × 10^5.5^ TCID_50_ of La Sota NDV. After 12 h of infection, the monolayers were washed two times with PBS, digested by trypsin (0.001%; GIBCO) in DMEM at room temperature for 20 min, and then washed two times with PBS, the monolayers where incubated in DMEM without serum/antibiotics for 12 h. To detect SP ability to inhibit fusion and further cell-cell viral spread, they were added to the medium before or after trypsin digestion. Monolayers were fixed with 3:1 methanol:acetone and stained with 1% crystal violet.

### 3.7. Statistical Analysis

Data analysis was performed by PASW 18 software (PASW 18.0 (2009), Chicago, IL, USA). All variables were tested in triplicate for each experiment and experiments were repeated at least three times. The CC_50_ values were determined by Probit regression analysis. One- way ANOVA with Dunnet’s post hoc test was used for comparisons *vs.* viral control. Values were expressed as the mean ± standard deviation (SD). The results of the comparisons *vs.* viral control were considered significantly different if *p* < 0.05 or highly significantly different if *p* < 0.001.

## 4. Conclusions

Characterization by chemical analysis indicates that the structure of the obtained sulphated polysaccharide is similar to that of ulvan-rich extracts already reported in the literature.

The SP and their mixture inhibit NDV *in vitro* and they did not exhibit significant toxicity at effective concentrations. Ulvan exerted no direct inactivating effect on virions in virucidal assay. Based on our findings from syncytia reduction and cell-cell fusion inhibition assays, we support the concept that ulvan acts at the same stages of the infection as fucoidan. The polygonogram and the median-effect plot show that ulvan acts as an antagonist of fucoidan in their mixture at the syncytia reduction assay, probably because both act on the same target.

Our results indicate that ulvan inhibits syncytia formation only if it is added before F protein cleavage (before trypsin digestion). This suggests that ulvan inhibits viral fusion by interacting with the intact F0 protein but not with the mature F protein. Ulvan alone showed better anti cell-cell spread activity than fucoidan, and their mixture apparently exhibited more potent effect.

Understanding the mechanism of antiviral activity of brown and green algae SPs is important for their future development as antiviral drugs for clinical or veterinary use. Nevertheless, it would be very useful to search for natural compounds that act on different targets along the viral infection process (perhaps other compounds from seaweed) and in a synergy with ulvan or fucoidan, in such a way to obtain a more effective combination therapy, which could exhibit a viral suppression and lower resistance than a single compound.

## References

[1] Gerber P., Dutcher J.D., Adams E.V., Sherman J.H. (1958). Protective effect of seaweed extracts for chicken embryos infected with influenza B or mumps virus. Proc. Soc. Exp. Biol. Med..

[2] Damonte E.B., Matulewicz M.C., Cerezo A.S. (2004). Sulfated seaweed polysaccharides as antiviral agents. Curr. Med. Chem..

[3] Elizondo-Gonzalez R., Cruz-Suarez L.E., Ricque-Marie D., Mendoza-Gamboa E., Rodriguez-padilla C., Trejo-Avila L.M. (2012). *In vitro* characterization of the antiviral activity of fucoidan from *Cladosiphon okamuranus* against Newcastle Disease Virus. Virol. J..

[4] Trejo-Avila L.M., Morales-Martínez M.L., Ricque-Marie D., Cruz-Suarez L.E., Zapata-Benavides P., Morán-Santibañez K., Rodríguez-Padilla C. (2014). *In vitro* anti-canine distemper virus activity of fucoidan extracted from the brown alga *Cladosiphon okamuranus*. Virus Dis..

[5] Patarra R.F., Paiva L., Neto A.I., Lima E., Baptista J. (2011). Nutritional value of selected macroalgae. J. Appl. Phycol..

[6] Lahaye M., Kaeffer B. (1997). Seaweed dietary fibres: Structure, physico-chemical and biological properties relevant to intestinal physiology. Sci. Aliments.

[7] Lahaye M., Robic A. (2007). Structure and functional properties of ulvan, a polysaccharide from green seaweeds. Biomacromolecules.

[8] Alves A., Sousa R.A., Reis R.L. (2013). *In vitro* cytotoxicity assessment of ulvan, a polysaccharide extracted from green algae. Phytother. Res..

[9] Qi H., Liu X., Zhang J., Duan Y., Wang X., Zhang Q. (2012). Synthesis and antihyperlipidemic activity of acetylated derivative of ulvan from *Ulva pertusa*. Int. J. Biol. Macromol..

[10] Mendes G.S., Soares A.R., Martins F.O., Albuquerque M.C., Costa S.S., Yoneshigue-Valentin Y., Gestinari L.M., Santos N., Romanos M.T. (2010). Antiviral activity of green marine alga *Ulva fasciata* on the replication of human metapneumovirus. Rev. Inst. Med. Trop. Sao Paulo.

[11] Jaulneau V., Laffite C., Jacquet C., Fournier S., Salamagne S., Briand X., Esquerré- Tugayé M.T., Dumas B. (2010). Ulvan, a sulfated polysaccharide from green algae, activates plant immunity through the jasmonic acid signaling pathway. J. Biomed. Biotechnol..

[12] Tabarsa M., Han J.H., Kim C.Y., You S.G. (2012). Molecular characteristics and immunomodulatory activities of water soluble sulfated polysaccharides from *Ulva pertusa*. J. Med. Food.

[13] Leiro J.M., Castro C., Arranz J.A., Lamas J. (2007). Immunomodulating activities of acidic sulphated polysaccharides obtained from the seaweed *Ulva rigida* C. Agardh. Int. Immunopharmacol..

[14] Mao W., Zang X., Li Y., Zhang H. (2006). Sulfated polysaccharides from marine green algae *Ulva conglobata* and their anticoagulant activity. J. Appl. Phycol..

[15] Peña-Rodríguez A., Mawhinney T.P., Ricque-Marie D., Cruz-Suárez L.E. (2011). Chemical composition of cultivated seaweed *Ulva clathrata* (Roth) C. Agardh. Food Chem..

[16] Robic A., Sassi J-F., Lahaye M. (2008). Impact of stabilization treatments of the green seaweed *Ulva rotundata* (Chlorophyta) on the extraction yield, the physico-chemical and rheological properties of ulvan. Carbohydr. Polym..

[17] Tabatabai M.A. (1974). Determination of sulfate in water samples. Sulphur. Inst..

[18] Quemener B., Marot C., Mouillet L., da Riz V., Diris J. (2000). Quantitative analysis of hydrocolloids in food systems by methanolysis coupled to reverse HPLC. Part 1. Gelling carrageenans. Food Hydrocoll..

[19] Robic A., Rondeau-Mouro C., Sassi J.-F., Lerat Y., Lahaye M. (2009). Structure and interactions of ulvan in the cell wall of the marine green algae *Ulva rotundata* (Ulvales, Chlorophyceae). Carbohydr. Polym..

[20] Miller P.J., Decanini E.L., Afonso C.L. (2010). Newcastle disease: Evolution of genotypes and the related diagnostic challenges. Infect. Genet. Evol..

[21] Mayo M.A. (2002). Virus taxonomy-Houston 2002. Arch. Virol..

[22] Lamb R.A., Parks G.D., Knipe D.M., Howley P.M. (2007). Paramyxoviridae: The viruses and their replication. Fields Virology.

[23] Taboada C., Millán R., Miguez I. (2010). Composition, nutritional aspects and effect on serum parameters of marine algae *Ulva rigida*. J. Sci. Food Agric..

[24] Yaich H., Garna H., Besbes S., Paquot M., Blecker C., Attia H. (2013). Effect of extraction conditions on the yield and purity of ulvan extracted from *Ulva lactuca*. Food Hydrocoll..

[25] Quemener B., Lahaye M. (1997). Sugar determination in ulvans by a chemical-enzymatic method coupled to high performance anion exchange chromatography. J. Appl. Phycol..

[26] DeReviers B., Leproux A. (1993). Characterization of polysaccharides from *Enteromorpha intestinalis* (L.) Link. Chlorophyta. Carbohydr. Polym..

[27] Percival E., McDowell R.H. (1967). Chemistry and Enzymology of Marine Algal Polysaccharides.

[28] Ray B., Lahaye M. (1995). Cell-wall polysaccharides from the marine green alga *Ulva “rigida”* (Ulvales Chlorophyta)-Chemical structure of ulvan. Carbohydr. Res..

[29] Robic A., Bertrand D., Sassi J.F., Lerat Y., Lahaye M. (2009). Determination of the chemical composition of ulvan, a cell wall polysaccharide from *Ulva* spp. (Ulvales, Chlorophyta) by FT-IR and chemometrics. J. Appl. Phycol..

[30] Hernández-Garibay E., Zertuche-González J.A., Pacheco-Ruíz I. (2010). Isolation and chemical characterization of algal polysaccharides from the green seaweed *Ulva clathrata* (Roth) C. Agardh. J. Appl. Phycol..

[31] Pengzhan Y., Quanbin Z., Ning L., Zuhong X., Yanmei W., Zhi’en L. (2003). Polysaccharides from *Ulva pertusa* (Chlorophyta) and preliminary studies on their antihyperlipidemia activity. J. Appl. Phycol..

[32] Chiellini F., Morelli A., Pignatello R. (2011). Ulvan: A Versatile Platform of Biomaterials from Renewable Resources. Biomaterials—Physics and Chemistry.

[33] Song X., Yin Z., Zhao X., Cheng A., Jia R., Yuan G., Xu J., Fan Q., Dai S., Lu H. (2013). Antiviral activity of sulfated *Chuanmingshen violaceum* polysacchare against Newcastle disease virus. J. Gen. Virol..

[34] Ivanova V., Rouseva R., Kolarova M., Serkedjieva J., Rachev R., Manolova N. (1994). Isolation of a polysaccharide with antiviral effect from *Ulva lactuca*. Prep. Biochem..

[35] Chiu Y.H., Chan Y.L., Li T.L., Wu C.J. (2012). Inhibition of Japanese encephalitis virus infection by the sulfated polysaccharide extracts from *Ulva lactuca*. Mar. Biotechnol. (N.Y.).

[36] Lee E., Pavy M., Young N., Freeman C., Lobigs M. (2006). Antiviral effect of the heparan sulfate mimetic, PI-88, against dengue and encephalitic flaviviruses. Antivir. Res..

[37] Hayashi T., Hayashi K., Kanekiyo K., Ohta Y., Lee J.B., Torrence P.F. (2007). Promising antiviral Glyco-molecules from an edible alga. Combating the Threat of Pandemic Influenza: Drug Discovery Approaches.

[38] Vlieghe P., Clerc T., Pannecouque C., Witvrouw M., de Clercq E., Salles J.P., Kraus J.L. (2002). Synthesis of new covalently bound kappa-carrageenan-AZT conjugates with improved anti-HIV activities. J. Med. Chem..

[39] Takebe Y., Saucedo C.J., Lund G., Uenishi R., Hase S., Tsuchiura T., Kneteman N., Ramessar K., Tyrrell D.L., Shirakura M. (2013). Antiviral lectins from red and blue-green algae show potent *in vitro* and *in vivo* activity against hepatitis C virus. PLoS One.

[40] Chou T.C. (2006). Theoretical basis, experimental design, and computerized simulation of synergism and antagonism in drug combination studies. Pharmacol. Rev..

[41] Chou T.C., Talalay P. (1984). Quantitative analysis of dose-effect relationships: The combined effects of multiple drugs or enzyme inhibitors. Adv. Enzyme Regul..

[42] Feng J.Y., Ly J.K., Myrick F., Goodman D., White K.L., Svarovskaia E.S., Borroto-Esoda K., Miller M.D. (2009). The triple combination of tenofovir, emtricitabine and efavirenz shows synergistic anti-HIV-1 activity *in vitro*: A mechanism of action study. Retrovirology.

[43] Klein M.B., Campeol N., Lalonde R.G., Brenner B., Wainberg M.A. (2003). Didanosine, interferon-alfa and ribavirin: A highly synergistic combination with potential activity against HIV-1 and hepatitis C virus. AIDS.

[44] Yang Z.H., Crouch J.Y., Chou T.C., Hsiung G.D. (1990). Combined antiviral effects of paired nucleosides against guinea pig cytomegalovirus replication *in vitro*. J. Antivir. Res..

[45] Damonte E.B., Matulewicz M.C., Cerezo A.S., Coto C. (1996). Herpes simplex virus inhibitory sulfated xylogalactans from the red seaweed *Nothogenia fastigiata*. Chemotherapy.

[46] Mandal P., Pujol C.A., Carlucci M.J., Chattopadhyay K., Damonte E.B., Ray B. (2008). Anti-herpetic activity of a sulfated xylomannan from *Scinaia hatei*. Phytochemistry.

[47] Bouhlal R., Haslin C., Chermann J.-C., Colliec-Jouault S., Sinquin C., Simon G., Cerantola S., Riadi H., Bourgougnon N. (2011). Antiviral activities of sulfated polysaccharides isolated from *Sphaerococcus coronopifolius* (Rhodophytha, Gigartinales) and *Boergeseniella thuyoides* (Rhodophyta, Ceramiales). Mar. Drugs.

[48] Paskaleva E.E., Lin X., Li W., Cotter R., Klein M.T., Roberge E., Yu E.K., Clark B., Veille J.C., Liu Y. (2006). Inhibition of highly productive HIV-1 infection in T cells, primary human macrophages, microglia, and astrocytes by *Sargassum fusiforme*. AIDS Res. Ther..

[49] Nyberg K., Ekblad M., Bergström T., Freeman C., Parish C.R., Ferro V., Trybala E. (2004). The low molecular weight heparan sulfate-mimetic, PI-88, inhibits cell-to-cell spread of herpes simplex virus. Antivir. Res..

[50] Cagno V., Donalisio M., Civra A., Volante M., Veccelli E., Oreste P., Rusnati M., Lembo D. (2014). Highly sulfated K5 *Escherichia coli* polysaccharide derivatives inhibit respiratory syncytial virus infectivity in cell lines and human tracheal-bronchial histocultures. Antimicrob. Agents Chemother..

[51] Tako M., Yoza E., Thoma S. (2000). Chemical characterization of acetyl fucoidan and alginate from commercially cultured *Cladosiphon okamuranus*. Bot. Mar..

[52] Moll B. (2004). Aquatic Surface Barriers and Methods for Culturing Seaweed.

[53] Cunniff P., AOAC (1997). Method 984.13. Official Methods of Analysis.

[54] Reed L.J., Muench H. (1938). A simple method of estimating fifty percent endpoints. Am. J. Hyg..

[55] Zhu J., Jiang X., Liu Y., Tien P., Gao G.F. (2005). Design and characterization of viral polypeptide inhibitors targeting Newcastle disease virus fusion. J. Mol. Biol..

